# Cytotoxic Tumour-Selective 1,5-Diaryl-3-Oxo-1,4-Pentadienes Mounted on a Piperidine Ring

**DOI:** 10.3390/medicines8120078

**Published:** 2021-12-16

**Authors:** Praveen K. Roayapalley, Hiroshi Sakagami, Keitaro Satoh, Shigeru Amano, Kenjiro Bandow, Renato J. Aguilera, Karla G. Cano Hernandez, Austre Y. Schiaffino Bustamante, Stephen G. Dimmock, Rajendra K. Sharma, Umashankar Das, Jonathan R. Dimmock

**Affiliations:** 1 Drug Discovery and Development Research Group, University of Saskatchewan, Saskatoon, SK S7N 5E5, Canada; umashankar.usask@gmail.com (U.D.); jr.dimmock@usask.ca (J.R.D.); 2 School of Dentistry, Meikai University, Sakado, Saitama 350-0283, Japan; sakagami@dent.meikai.ac.jp (H.S.); k-satoh@dent.asahi-u.ac.jp (K.S.); shigerua@dent.meikai.ac.jp (S.A.); kbando@dent.meikai.ac.jp (K.B.); 3 Department of Biological Sciences and Border Biomedical Research Center, The University of Texas at El Paso, El Paso, TX 79968-0519, USA; raguilera@utep.edu (R.J.A.); Karla.CanoHernandez@UTSouthwestern.edu (K.G.C.H.); aschiaf@emory.edu (A.Y.S.B.); 4 Department of Finance, National University of Singapore, Singapore 119245, Singapore; dimmock@nus.edu.sg; 5 Department of Pathology and Laboratory Medicine, College of Medicine, University of Saskatchewan, Saskatoon, SK S7N 0W8, Canada; rajendra.sharma@usask.ca

**Keywords:** 4-piperidones, unsaturated ketones, cytotoxicity, quantitative structure-activity relationships (QSAR), Western blot, cell cycle inhibition

## Abstract

A series of 3,5-bis(benzylidene)-4-piperidones **2a**–**u** were prepared as candidate cytotoxic agents. In general, the compounds are highly toxic to human gingival carcinoma (Ca9-22), human squamous carcinoma-2 (HSC-2) and human squamous carcinoma-4 (HSC-4) neoplasms, but less so towards non-malignant human gingival fibroblast (HGF), human periodontal ligament fibroblast (HPLF) and human pulp cells (HPC), thereby demonstrating tumour-selective toxicity. A further study revealed that most of the compounds in series **2** were more toxic to the human Colo-205 adenocarcinoma cell line (Colo-205), human HT29 colorectal adenocarcinoma cells (HT-29) and human CEM lymphoid cells (CEM) neoplasms than towards non-malignant human foreskin Hs27 fibroblast line (Hs27) cells. The potency of the cytotoxins towards the six malignant cell lines increased as the sigma and sigma star values of the aryl substituents rose. Attempts to condense various aryl aldehydes with 2,2,6,6-tetramethyl-4-piperidone led to the isolation of some 1,5-diaryl-1,4-pentadien-3-ones. The highest specificity for oral cancer cells was displayed by **2e** and **2r**. In the case of **2r**, its selective toxicity exceeded that of doxorubicin and melphalan. The enones **2k**, **m**, o have the highest SI values towards colon cancer and leukemic cells. Both **2e**,**r** inhibited mitosis and increased the subG1 population (with a transient increase in G2/M phase cells). Slight activation of caspase-3, based on the cleavage of poly(ADP-ribose)polymerase (PARP) and procaspase 3, was detected.

## 1. Introduction

**Figure 1:**
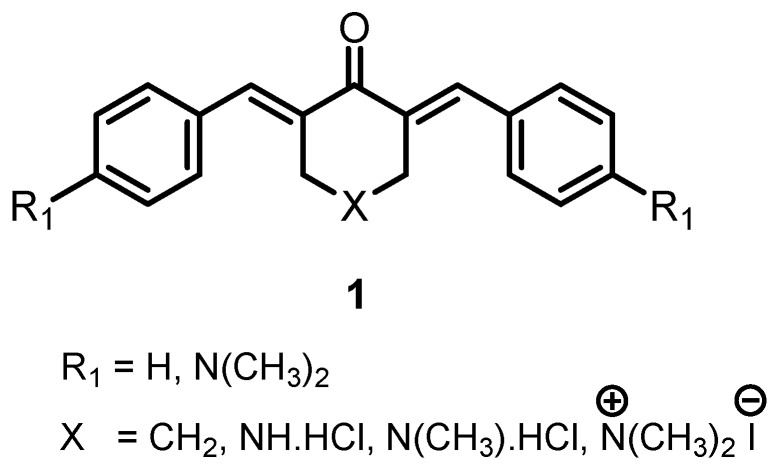
Initial series of cytotoxins **1**.

A major interest in our laboratories is the design, synthesis and antineoplastic evaluation of 1,5-diaryl-3-oxo-1,4-pentadienes mounted on a variety of scaffolds, which give rise to series **1** (Figure 1). Most previous studies of structurally-related compounds were conducted without or with very low numbers of normal cells at the same time as cancer cell lines, making it difficult to compare tumor-specificity between compounds [1,2,3,4,5,6,7,8,9,10]. Among these previous 10 papers, three papers are from our research group [5,6,7]. Our group used four human oral squamous cell carcinoma cell lines (Ca9-22, HSC-2, HSC-3, HSC-4) with three human normal oral mesenchymal cells (HGF, HPLF, HPC) [5,6] and human CRL1790 (non-malignant colon cells) [10]. Other groups have used human HepG2 (hepatocellular carcinoma), HeLa (cervical cancer), K562 (erythroleukemia), THP-1 (monocytic leukemia), LO2 (hepatocyte cell line) [1], HCT-116 (colorectal carcinoma), MCF-7 (breast adenocarcinoma) [2], HepG2, MCF-7 [3], GCIY (stomach cancer), HCT-116, DLD1 (colorectal adenocarcinoma), SW680 (colon cancer), A549 (lung carcinoma), PK1 (pig kidney epithelial cells), ACHN (renal adenocarcinoma), HUH7 (immortal cell line composed of epithelial-like, tumorigenic cells), OVK18 (ovarian cancer), MCF-7, 8505c (thyroid carcinomas), G361 (melanoma) and PC3 (prostate cancer) [4], as well as A2780 (ovarian cancer), ACHN, HCT-116 and PC-3 and U937-GTB (leukemic monocyte lymphoma) cell lines [8], B16 (murine melanoma) and L1210 (murine lymphoma) cells [7] and P-388 (murine leukemia) [8] cell lines. Thus, very few previous studies have used human oral cancer cells. Furthermore, most of these papers, except [1], have not used appropriate normal control cells derived from the same tissues at the same time. By comparing the cytotoxicity of a total 21 compounds (**2a**–**2u**) against oral malignant and non-malignant cells, with two reference compounds, the present study demonstrated that **2r** showed the highest tumor-specificity. One of the purposes of the present study is to prepare compounds which are easy to synthesize and have selective toxicity against oral cancer, for use in a series of our research projects for dental application [11,12].

**Figure 2:**
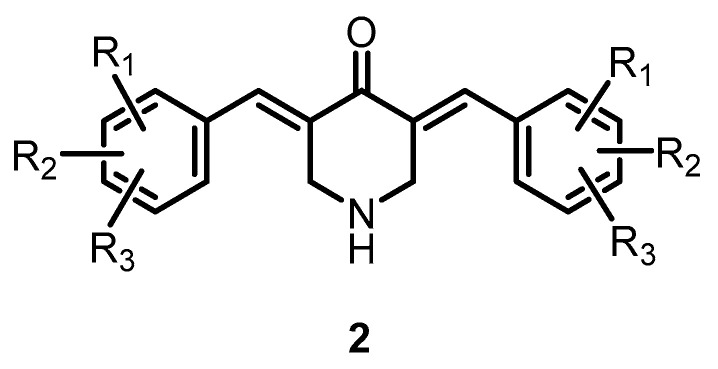
The general structure of the compounds in series **2.** The aryl substituents are indicated in Tables 1, 2 and 4.

Recently, we have concentrated our efforts on 4-piperidones which have the general structure **2** as indicated in Figure 2. First, their bioactivity is likely due to reactivity with cellular thiols. However, these dienones do not react readily, if at all, with amino and hydroxyl groups [13,14], which are present in nucleic acids. Thus, the possibility of these unsaturated ketones causing genotoxic effects, which are present in a number of anticancer drugs, is reduced. An important goal of this investigation was to find compounds which are more cytotoxic to neoplasms than towards non-malignant cells. If this objective is achieved, then investigations were planned in order to find correlations between various physicochemical substituents and the magnitude of antineoplastic potencies. Such cytotoxic warheads could be converted into more complex candidate anticancer agents through suitable molecular modifications. For example, the placement of a group onto the piperidyl nitrogen atom, which contains a positively charged phosphorous atom, may have a greater attraction to tumour mitochondria than to mitochondria of non-malignant cells [15].

## 2. Materials and Methods

### 2.1. Synthesis of Compounds

^1^H and ^13^C Nuclear Magnetic Resonance (NMR) spectra were obtained using a Bruker 500 MHz spectrometer equipped with a Broad Band Observe (BBO) probe. Chemical shifts (δ) are reported in ppm. Melting points were determined using a DigiMelt-MPA 160 instrument and are uncorrected. The mass spectra were obtained using a Jeol JMS-T100 GCv Accu tof-gcV4G instrument.

The general procedure for the syntheses of **2a**–**u** is as follows [16]. The aryl aldehyde (11.46 mmol) and 4-piperidone hydrochloride monohydrate (5.60 mmol) were added to glacial acetic acid (15 mL) and stirred for five minutes. Dry hydrogen chloride gas was passed through this mixture for about 45 min until a clear solution was obtained. The reaction mixture was stirred at room temperature for 24 h, and the precipitate formed was collected by filtration. The crude product was treated with a mixture of 10 mL of saturated aqueous potassium carbonate solution (25% *w*/*v*) and 10 mL of acetone and stirred at room temperature for 45 min. The free base was collected by filtration, washed with ice-cold water and dried. The compounds were taken for the next reaction with the acid chloride without further purification.

The percentage yields, melting points, ^1^H and ^13^C NMR spectra and mass spectra of **2a**–**u** are given in the Supplemental section of this paper.

**Figure 3:**
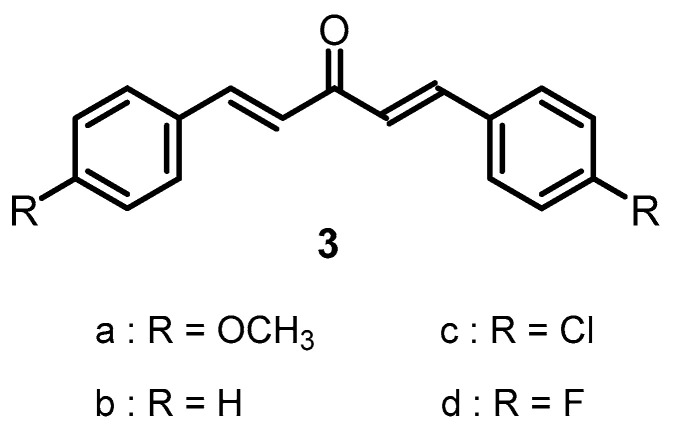
Products of the reaction between 2,2,6,6-tetramethyl-4-piperidone and various aryl aldehydes, **a:** R = OCH_3_; **b:** R = H; **c:** R = Cl; **d:** R = F.

The general method adopted for the formation of **3a**–**d** (Figure 3) is as follows. The aryl aldehyde (2.64 mmol) and 2,2,6,6-tetramethyl-4-piperidone (1.28 mmol) were dissolved in 20 mL of methanol (**3a**) and absolute ethanol (**3b**–**d**) and stirred at room temperature for 5 min. Sodium metal (4.5 mmol) was added slowly to the reaction mixture at 0 °C. After all the sodium metal dissolved, the reaction mixture was stirred at room temperature for 1 h and then heated to 50–55 °C overnight. The reaction mixture was cooled to 5–10 °C, and 5 mL 6N hydrochloric acid was slowly added. The organic solvent was completely evaporated, and the residue was extracted with ethyl acetate. The organic extract was dried over anhydrous sodium sulfate and concentrated to attain the crude compound, which was purified by column chromatography using silica gel-60 (0.040–0.063 mm, 230–400 mesh) and 5% ethyl acetate in hexane as the mobile phase.

The percentage yields, melting points, ^1^H NMR spectra and mass spectra of **3a**–**d** and ^13^C NMR spectra of **3b**–**d** are presented in the Supplemental section of this article.

### 2.2. QSAR Studies

The sigma/sigma star (σ/σ*), pi (π) and Molar Refractivity (MR) values used in the QSAR studies were taken from a reference source [17,18]. The linear and semilogarithmic plots were undertaken using a Statistical Package for the Social Sciences (SPSS) package [19].

### 2.3. Cytotoxicity Assays

A literature procedure was followed when evaluating series **2** against Ca9-22, HSC-2, HSC-4, HGF, HPLF and HPC cells except that the cells were incubated with the compounds for 48 h, not 24 h [20]. In brief, varying amounts of material were incubated at 37 °C with the neoplastic and non-malignant cells in Dulbecco’s Modified Eagle Medium (DMEM) supplemented with 10% heat-inactivated fetal bovine serum. The cell viability was determined by the 3-(4,5-dimethylthiazol-2-yl)-2,5-diphenyl-2H-tetrazolium bromide (MTT) method, as described previously [20]. We have previously reported that doxorubicin-induced potent cytotoxicity against human normal oral epithelial cells such as human oral keratinocyte (HOK) and primary human gingival epithelial cells (HGEP) [21]. Therefore, we used human normal oral mesenchymal cells, rather than epithelial cells, to develop new anticancer drugs that have much lower keratinocyte toxicity [22]. Furthermore, HGF and Ca9-22 were derived from gingival tissue.

The method used in the evaluation of series **2** against Colo205, HT-29, CRM and Hs27 cells has been described previously [23,24]. In brief, the cells were cultured in DMEM (Colo205, HT29 and Hs27) or RPMI −1640 (CEM) media supplemented with 10% heat-inactivated fetal bovine serum. Different concentrations of the compounds were added to the cell cultures and then viability was measured using the Differential DNA Staining (DNS) assay after 24 h incubation [23,24]. After analysis of the effects of a range of concentrations of the compounds, the CC_50_ value (the concentration of the compound to kill 50% of the cells) was determined. After subtracting the percentage of cell death caused by the solvent (DMSO-treated cells), a linear interpolation equation was used to determine the CC_50_ values [24,25].

The induction of apoptosis in Ca9-22 cells was evaluated through morphological changes, and the Western blot and cell cycle analysis has been described previously [26]. In brief, cell morphology was checked periodically under the light microscope (EVOS FL, ThermoFisher Scientific, Waltham, MA, USA). Western blot analysis was performed as described previously [27]. Briefly, cells were collected and lysed, and protein samples of cell lysates (9 µg) were applied to SDS-polyacrylamide gel electrophoresis. After electrophoresis, the separated proteins were transferred onto a PVDF filter. The blots were blocked in skim milk and then probed for 120 min with a primary antibody cocktail (1:250) from an Apoptosis Western Blot Cocktail kit (Abcam, Cambridge, UK). The cocktail included anti-caspase 3, anti-PARP and anti-actin antibodies. The blots were washed and then probed with a horseradish peroxidase-conjugated secondary antibody cocktail (1:100) from the kit. The cocktail included anti-mouse and anti-rabbit secondary antibodies. The immunoreactivities were determined using Amersham ECL Select (Cytiva, Tokyo, Japan). Images were acquired using the ChemiDoc MP System and Image Lab 4.1 software (Bio-Rad Laboratories, Hercules, CA, USA).

For cell cycle analysis, cells were fixed with 1% paraformaldehyde, treated with 0.2 mg/mL RNase A, stained with 0.01% propidium iodide, filtered through Falcon^®^ cell strainers (40 µM) (Corning, NY, USA), subjected to cell sorting and then analyzed (SH800 Series; SONY Imaging Products and Solutions Inc., Kanagawa, Japan).

## 3. Results

The compounds in series **2** were prepared by the reaction of 4-piperidone with various aryl aldehydes. These dienones were screened against human Ca9-22, HSC-2 and HSC-4 oral squamous carcinomas (Table 1), as well as human gingival fibroblast (HGF), human periodontal ligament fibroblasts (HPLF) and human pulp cells (HPC) (Table 2 and Table 3). In addition, the evaluation of series **2** was also assessed against human Colo205 and HT-29 colon cancers, CEM lymphoid leukemia cells and human Hs27 foreskin fibroblasts (Table 4 and Table 5).

**Figure 4:**
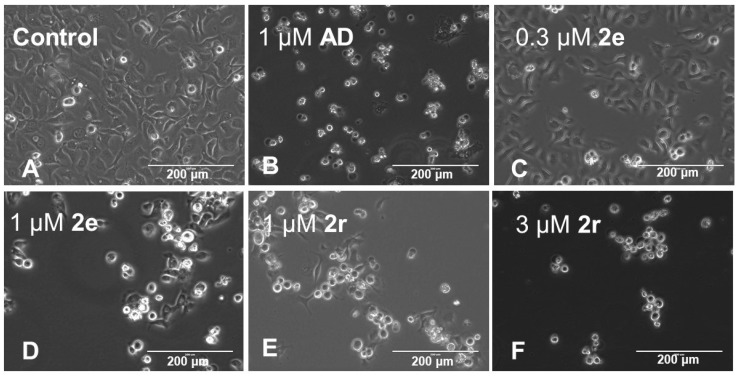
Morphological change induced in Ca9-22 cells after 24 h incubation without (control) (**A**); with 1 μM actinomycin D (**AD**, reference compound) (**B**); with 0.3 and 1 μM of **2e** (**C**,**D**); and with 1 and 3 μM of **2r** (**E**,**F**). Actinomycin D induced apoptotic bodies, whereas **2e** and **2r** induced cell spreading at lower concentrations and cell shrinkage at higher concentrations (The scale bar is 200 μm). This point was clarified by Western blot analysis (Figure 5) and cell cycle analysis (Table 6).

**Figure 5:**
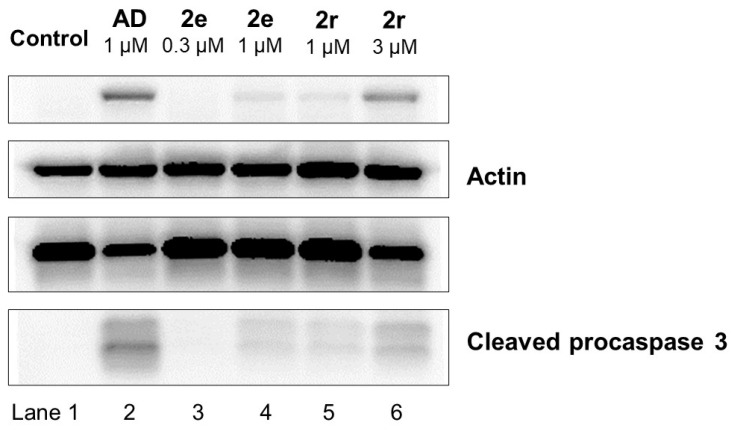
Western blot analysis of **2e**, **r**. Ca9-22 cells were incubated for 24 h without (control) and with the indicated concentrations of samples, and cell lysates were prepared as described in Materials and Methods. Protein(9 μg) was loaded to each lane for Western blot analysis, and images after long exposure with contrast adjustment are presented. Raw data of stained gel and images after short and long exposure (without and with contrast adjustment) are shown in Supplementary Figure S1.

Investigations were implemented to find some of the ways that two representative compounds exert their cytotoxic potencies. Control Ca9-22 cells show a homogenous population (Figure 4A), with no detectable amount of cleaved products of PARP and procaspase-3 (substrates of caspase 3) (lane 1 in Figure 5) and low subG1 population levels (consisting of fragmented DNA) (1.7%) (Table 6). When Ca9-22 cells were treated for 24 h with 1 μM actinomycin D (reference compound), apparent induction of apoptosis markers such as cell shrinkage (Figure 4B), cleavage of PARP and procaspase (lane 2 in Figure 5) and subG1 population accumulation (Table 6) were confirmed. Pictures of the morphologies of the cells before the cell harvest are shown in the lower panel of Supplementary Figure S2. Reproducible morphological changes were confirmed.

When cells were treated with a lower concentration of **2e** (0.3 μM), slight cell speading (Figure 4C), an undetectable level of cleaved products (lane 3 in Figure 5) and minor levels of subG1 (2.2%) (Table 6) were observed. When **2e** concentration was increased to 1 μM, cell shrinkage (Figure 4D), cleavage products (lane 4 in Figure 5) and subG1 accumulation (9.3%) were more apparent. A similar magnitude of changes in these markers was observed in the cells treated with **2r** (1 μM) (Figure 4E, lane 5 in Figure 5 and Table 6). It should be noted that the percentage of G2/M was the highest in the cells treated with **2e** (1 μM) and **2r** (1 μM) (50.2 and 57.9%, respectively) (Table 6). When **2r** concentration was increased up to 3 μM, more marked cell shrinkage (Figure 4F), caspase-3 activation (cleavage of PARP and procaspase) (lane 6 in Figure 5) and subG1 accumulation (24.5%), along with a decline in G2/M (29.1%) (Table 6), were observed. These data suggest that **2e** and **2r** induced G2/M accumulation and then subG1 or G1 accumulation.

In an attempt to prepare 3,5-bis(4-methoxybenzylidene)-2,2,6,6-tetramethyl-4-piperidone, the product obtained was 1,5-bis(4-methoxyphenyl)-3-oxo-1,4-pentadiene. This reaction was shown to be a general method for preparing these acyclic derivatives, and a reaction mechanism for their formation was proposed.

## 4. Discussion

The compounds in series **2** were prepared by acidic catalysis of a number of aryl aldehydes with 4-piperidone using a literature procedure [16]. The average yield is 72%. All of the compounds in series **2** were characterized by ^1^H and ^13^C NMR spectroscopy as well as mass spectrometry, and these details may be found in the Supplemental Section of this article.

A number of different groups are present in the aryl rings of **2a**–**u** as indicated in Table 1. In particular, methoxy and fluoro groups are present since they have been incorporated into a number of potent cytotoxins prepared in our laboratory [10,28,29].

**Table 1:** Evaluation of **2a**–**u** against Ca9-22, HSC-2 and HSC-4 human oral squamous cell carcinoma cell lines.

Human Oral Squamous Cell Carcinoma Cell Lines									
		Ca9-22		HSC-2		HSC-4		Average	
Compound	R	CC_50_ (µM) ^a^	SI ^b^	CC_50_ (µM) ^a^	SI ^b^	CC_50_ (µM) ^a^	SI ^b^	CC_50_ (µM) ^a^	SI ^b^
**2a** ^c^	H	0.27 ± 0.03	58.1	0.31 ± 0.05	50.6	0.41 ± 0.13	38.3	0.33 ± 0.07	49.0
**2b**	2-OCH_3_	0.13 ± 0.02	40.3	0.33 ± 0.09	15.9	0.50 ± 0.04	10.5	0.32 ± 0.18	22.2
**2c** ^d^	3-OCH_3_	0.10 ± 0.03	18.2	0.02 ± 0.04	91.0	0.12 ± 0.02	15.2	0.08 ± 0.01	41.5
**2d** ^e^	4-OCH_3_	3.13 ± 0.40	17.8	7.97 ± 0.68	6.98	6.86 ± 0.77	8.10	5.99 ± 2.53	11.0
**2e** ^e^	3,4-(OCH_3_)_2_	0.02 ± 0.01	136	0.24 ± 0.04	11.3	0.07 ± 0.00	38.9	0.11 ± 0.11	62.1
**2f** ^d^	2,5-(OCH_3_)_2_	0.06 ± 0.01	23.3	0.17 ± 0.07	8.23	0.18 ± 0.08	7.78	0.14± 0.07	13.1
**2g** ^e^	2,4,6-(OCH_3_)_3_	6.90 ± 0.30	5.00	7.95 ± 0.70	4.34	8.93 ± 0.75	3.86	7.93 ± 1.08	4.40
**2h** ^e^	3,4,5-(OCH_3_)_3_	0.04 ± 0.01	24.3	0.08 ± 0.01	12.1	0.11 ± 0.03	8.82	0.08 ± 0.04	15.1
**2i** ^d^	3,4-OCH_2_O	0.16 ± 0.07	102	0.53 ± 0.12	30.8	0.71 ± 0.22	23.0	0.47 ± 0.28	51.9
**2j** ^e^	2-F	0.16 ± 0.04	21.4	0.33± 0.23	10.4	0.23 ± 0.01	14.9	0.24 ± 0.09	15.6
**2k**	3-F	0.07 ± 0.01	26.3	0.13 ± 0.03	14.2	0.12 ± 0.05	15.3	0.11 ± 0.03	18.6
**2l** ^c^	4-F	0.14 ± 0.06	32.9	0.94 ± 1.28	4.90	0.16 ± 0.02	28.8	0.41± 0.45	22.2
**2m**	3,4-F_2_	0.06 ± 0.03	41.2	0.14 ± 0.05	17.6	0.07 ± 0.01	35.3	0.09 ± 0.04	31.4
**2n**	2,6-F_2_	0.17 ± 0.02	24.5	0.40 ± 0.10	10.4	0.31 ± 0.21	13.5	0.29 ± 0.11	16.1
**2o**	2-CH_3_	0.45 ± 0.04	26.4	0.79 ± 0.19	15.1	1.05 ± 0.34	11.3	0.76 ± 0.30	17.6
**2p**	2-NO_2_	0.13 ± 0.01	20.8	0.31 ± 0.06	8.71	0.30 ± 0.15	9.00	0.25 ± 0.10	12.8
**2q**	3-OCH_3,_ 4-OH	0.61 ± 0.23	13.4	0.61± 0.08	13.4	0.63 ± 0.16	13.0	0.62 ± 0.01	13.3
**2r**	3-OH, 4-OCH_3_	0.23 ± 0.01	214	0.29 ± 0.06	170	0.31 ± 0.12	159	0.28 ± 0.04	181
**2s** ^e^	4-OH	1.67 ± 0.13	54.1	2.52 ± 0.19	35.9	3.20 ± 0.58	28.3	2.46 ± 0.77	39.4
**2t** ^d^	3-OH	0.18 ± 0.01	28.2	0.21 ± 0.01	24.1	0.20 ± 0.04	25.4	0.20 ± 0.01	25.9
**2u**	2-Cl	0.10 ± 0.04	49.6	0.16 ± 0.02	31.0	0.25 ± 0.12	19.8	0.17 ± 0.08	33.5
Doxorubicin		0.24 ± 0.04	31.5	0.07 ± 0.00	108	0.08 ± 0.01	94.5	0.13 ± 0.10	78.0
Melphalan		27.4 ± 6.40	6.30	13.9 ± 3.80	12.4	14.4 ± 1.70	12.0	18.6 ± 7.63	10.2

^a^ The CC_50_ values are the concentrations of compounds required to kill 50% of the cells. ^b^ The letters SI refer to the selectivity index. The SI figures are the ratios of the average CC_50_ value of the compounds towards non-malignant HGF, HPLF and HPC cells (Table 2) and the CC_50_ figure of a compound against a specific neoplastic cell line. ^c^ Reference [16], ^d^ Reference [7], ^e^ Reference [30].

The cytotoxic properties of the compounds in series **2** were evaluated in two independent laboratories. In the first place, **2a**–**u** were screened against human Ca9-22, HSC-2 and HSC-4 squamous cell carcinomas, and the results are portrayed in Table 1. No less than 86% of the CC_50_ figures are submicromolar and 14% of the compounds have double-digit nanomolar CC_50_ values. The dienones with average CC_50_ values of less than 0.1 µM are as follows (with aryl substituents in parentheses): **2c** [3-OCH_3_], **2h** [3,4,5-(OCH_3_)_3_] and **2m** [3,4-F_2_]. These three compounds are equipotent with doxorubicin and are much more toxic than melphalan.

There are significant differences between several pairs of structural isomers. Thus, the mean CC_50_ value of **2c** is 75 times lower than the figure for **2d**. A similar comparison between **2g** and **2h** indicates a 99-fold difference in potency, while the mean CC_50_ figure for **2t** is 12 times lower than the value for **2s**.

**Table 2:** Evaluation of **2a**–**u** against HGF, HPLF and HSC human normal oral cell lines.

Human Normal Oral Cell Lines						
		HGF	HPLF	HPC	Average	PSE ^b^
Compound	R	CC_50_ (µM) ^a^	CC_50_ (µM) ^a^	CC_50_ (µM) ^a^	CC_50_ (µM) ^a^	
**2a** ^c^	H	7.78 ± 0.85	17.0 ± 2.29	22.3 ± 2.61	15.7 ± 7.33	14,849
**2b**	2-OCH_3_	2.11 ± 0.13	4.84 ± 3.52	8.77 ± 1.58	5.24 ± 3.35	6938
**2c** ^d^	3-OCH_3_	2.07 ± 0.11	2.47 ± 0.07	0.91 ± 0.08	1.82 ± 0.81	51,875
**2d** ^e^	4-OCH_3_	38.1 ± 12.4	>100	28.8 ± 0.52	>55.6 ± 38.7	>184
**2e** ^e^	3,4-(OCH_3_)_2_	2.06 ± 0.16	2.48 ± 0.28	3.61 ± 2.50	2.72 ± 0.80	56,455
**2f** ^d^	2,5-(OCH_3_)_2_	1.29 ± 0.60	2.01 ± 0.27	0.91 ± 0.02	1.40 ± 0.56	9357
**2g** ^e^	2,4,6-(OCH_3_)_3_	33.9 ± 8.27	48.0 ± 10.4	21.7 ± 1.72	34.5 ± 13.2	56
**2h** ^e^	3,4,5-(OCH_3_)_3_	1.13 ± 0.79	1.28 ± 0.71	0.51 ± 0.20	0.97 ± 0.40	18,875
**2i** ^d^	3,4-OCH_2_O	6.35 ± 0.12	7.91 ± 0.19	34.7 ± 12.2	16.3 ± 15.9	11,043
**2j** ^e^	2-F	4.40 ± 1.32	3.63 ± 0.57	2.24 ± 1.17	3.42 ± 1.09	6500
**2k**	3-F	2.05 ± 0.16	2.70 ± 0.12	0.78 ± 0.16	1.84 ± 0.98	16,909
**2l** ^c^	4-F	4.22 ± 1.31	7.07 ± 0.47	2.54 ± 0.14	4.61 ± 2.29	5415
**2m**	3,4-F_2_	1.68 ± 0.44	3.69 ± 0.64	2.04 ± 0.97	2.47 ± 1.07	34,889
**2n**	2,6-F_2_	2.07 ± 0.30	7.46 ± 0.38	2.97 ± 0.05	4.17 ± 2.89	5552
**2o**	2-CH_3_	5.58 ± 0.18	7.27 ± 0.21	22.8 ± 1.85	11.9 ± 9.51	2316
**2p**	2-NO_2_	1.71 ± 0.23	2.57 ± 0.07	3.82 ± 1.14	2.70 ± 1.06	5120
**2q**	3-OCH_3,_ 4-OH	5.76 ± 1.20	10.8 ± 1.10	8.08 ± 8.51	8.21 ± 2.50	2145
**2r**	3-OH, 4-OCH_3_	2.72 ± 0.18	54.6 ± 39.1	90.7 ± 16.2	49.3 ± 44.2	64,643
**2s** ^e^	4-OH	74.0 ± 6.56	>100	97.3 ± 4.62	>90.4 ± 14.3	>1602
**2t** ^d^	3-OH	2.23 ± 0.06	3.45 ± 0.30	9.53 ± 5.17	5.07 ± 3.91	12,950
**2u**	2-Cl	2.25 ± 0.15	5.07 ± 1.99	7.55 ± 1.62	4.96 ± 2.65	19,706
Doxorubicin		2.69 ± 0.35	>10.0	>10 ± 0.00	>7.56 ± 4.22	>60,000
Melphalan		148.0 ± 0.68	>200	169 ± 18.5	>172 ± 25.8	>55

^a^ The CC_50_ values are the concentrations of the compounds required to kill 50% of the cells. ^b^ The letters PSE refer to the potency-selectivity expression. These figures are the product of the reciprocal of the average CC_50_ values against Ca9-22, HSC-2 and HSC-4 cells and the average selectivity index (SI) value multiplied by 100. ^c^ Reference [16], ^d^ Reference [7], ^e^ Reference [30].

An important issue to resolve when considering the development of these compounds is whether they demonstrate tumour-selective toxicity. Consequently, the dienones in series **2** were evaluated against human HGF, HPLF and HPC non-malignant cells, and these data are presented in Table 2. Compounds causing the least toxicity, with average CC_50_ values greater than 30 µM, are **2d** [4-OCH_3_], **2g** [2,4,6-(OCH_3_)_3_], **2r** [3-OH-4-OCH_3_] and **2s** [4-OH]. On the other hand, compounds causing the most toxicity with an average CC_50_ value of less than 2 µM are **2c** [3-OCH_3]_, **2f** [2,5-(OCH_3_)_2_], **2h** [3,4,5-(OCH_3_)_3_] and **2k** [3-F].

The next phase of the investigation was to identify those compounds which demonstrate greater toxicity to neoplasms than towards non-malignant cell lines. Under clinical conditions, neoplasms are surrounded by a variety of normal cells. Hence in order to evaluate if these compounds demonstrate tumour-selective toxicity, selectivity index (SI) figures were generated which are the quotients of the average CC_50_ values towards HGF, HPLF and HPC cells and the CC_50_ value of the compound towards each neoplastic cell line. The data are presented in Table 1. The results indicate that the compounds in series **2** have SI values greater than 1; hence, they demonstrate tumour-selective toxicity. The ketones with average SI figures greater than 50 are **2e** [3,4-(OCH_3_)_2_], **2i** [3,4-OCH_2_O] and **2r** [3-OH,4-OCH_3_] and are clearly lead molecules. In particular, **2r** has a greater SI figure than doxorubicin.

Two properties that are very important in evaluating candidate cytotoxins are their potencies and selective toxicities. In order to identify compounds with both of these desirable features, potency-selectivity expression (PSE) values were obtained. These figures are the products of the reciprocal of the average CC_50_ value towards neoplastic cells multiplied by the average SI figure times 100. The PSE values of **2a**–**u** are presented in Table 2. Compounds which have PSE values over 25,000 are **2c**,**e**,**m**,**r**. The dienone **2r** has the highest PSE figure of 64,643, which is 30 times greater than the value of the structural isomer **2q**.

A summary of the identification of lead molecules is presented in Table 3. In particular, the 4-piperidone **2r** is identified as a promising compound in three of the four bioevaluations.

**Table 3:** Identification of lead molecules based on the data in Table 1 and Table 2.

Table	Bioevaluations	Promising Compounds
1	Average CC_50_ values	**2c**, **h**, **m**
1	Average SI figures	**2e**, **i**, **r**
2	Low toxicity to normal cells	**2d**, **g**, **r**, **s**
2	PSE	**2c**, **e**, **m**, **r**

The compounds in series **2** were also evaluated in a different laboratory. In this case, three human neoplastic cell lines were employed, namely, Colo205 and HT-29 colon cancers, as well as CEM leukemia cells. A non-malignant cell line, namely, human Hs27 fibroblasts, was also incorporated into the screens. The biodata generated are presented in Table 4.

**Table 4:** Evaluation of **2a**–**u** towards Colo205, HT-29, CEM and Hs27 cells.

		Human Tumour Cell Lines								Normal Cell Line	
		Colo205		HT-29		CEM		Average		Hs27	
Compound	R	CC_50_ (µM) ^a^	SI ^b^	CC_50_ (µM) ^a^	SI ^b^	CC_50_ (µM) ^a^	SI ^b^	CC_50_^a^	SI ^b^	CC_50_ (µM) ^a^	PSE ^c^
**2a** ^d^	H	10.7 ± 0.05	1.54	1.57 ± 0.21	10.5	9.29 ± 0.11	1.78	7.19	4.61	16.5 ± 0.35	64
**2b**	2-OCH_3_	1.32 ± 0.01	3.10	3.58 ± 0.21	1.14	0.82 ± 0.09	4.99	1.91	3.08	4.09 ± 0.56	161
**2c** ^e^	3-OCH_3_	0.32 ± 0.11	13.0	5.58 ± 0.52	0.75	4.51 ± 0.11	0.92	3.47	4.89	4.16 ± 0.32	141
**2d** ^f^	4-OCH_3_	14.0 ± 0.56	6.84	12.6 ± 0.00	7.60	4.45 ± 0.03	21.5	10.4	12.0	95.7 ± 0.11	115
**2e** ^f^	3,4-(OCH_3_)_2_	1.36 ± 0.17	0.65	3.46 ± 0.46	0.25	0.49 ± 0.02	1.80	1.77	0.90	0.88 ± 0.05	51
**2f** ^e^	2,5-(OCH_3_)_2_	0.36 ± 0.01	1.81	0.84 ± 0.01	0.77	0.42 ± 0.02	1.55	0.54	1.37	0.65± 0.02	254
**2g** ^f^	2,4,6-(OCH_3_)_3_	16.9± 0.23	1.63	9.63 ± 0.32	2.87	11.0 ± 1.06	2.51	12.5	2.34	27.6 ± 0.79	19
**2h** ^f^	3,4,5-(OCH_3_)_3_	0.27 ± 0.03	121	2.03 ± 0.00	16.1	0.31 ± 0.09	106	0.87	81.0	32.7 ± 0.56	9310
**2i** ^e^	3,4-OCH_2_O	8.84 ± 0.08	2.17	7.42± 1.33	2.59	1.88 ± 0.08	10.2	6.05	4.99	19.2 ± 2.38	83
**2j** ^f^	2-F	2.94 ± 0.33	0.68	3.46 ± 0.33	0.58	0.78 ± 0.04	2.55	2.39	1.27	1.99 ± 0.17	53
**2k**	3-F	4.19 ± 0.15	0.44	1.73 ± 0.15	1.06	0.21 ± 0.01	8.76	2.04	3.42	1.84 ± 0.32	168
**2l** ^d^	4-F	1.56 ± 0.28	1.21	5.51 ± 0.56	0.34	1.68 ± 0.25	1.13	2.92	0.89	1.89 ± 0.05	31
**2m**	3,4-F_2_	1.81 ± 0.31	16.1	3.50 ± 0.19	8.34	0.58 ± 0.05	50.3	1.96	24.9	29.2 ± 0.08	1270
**2n**	2,6-F_2_	2.22 ± 0.00	3.50	7.36 ± 0.88	1.06	7.88 ± 0.68	0.99	5.82	1.85	7.77 ± 0.89	32
**2o**	2-CH_3_	1.62 ± 0.11	30.4	3.54 ± 0.42	13.9	1.82 ± 0.10	27.1	2.33	23.8	49.3 ± 0.23	1022
**2p**	2-NO_2_	2.09 ± 0.33	11.6	2.76 ± 0.04	8.80	6.14 ± 0.56	3.96	3.66	8.12	24.3 ± 0.11	222
**2q**	3-OCH_3,_ 4-OH	1.73 ± 0.15	1.20	17.8 ± 1.48	0.12	1.92 ± 0.53	1.08	7.15	0.80	2.07 ± 0.07	11
**2r**	3-OH, 4-OCH_3_	4.58 ± 0.17	2.73	4.26 ± 0.01	2.93	4.04 ± 0.17	3.09	4.29	2.92	12.5 ± 1.61	68
**2s** ^f^	4-OH	4.17 ± 0.32	2.08	19.3 ± 0.48	0.45	1.99 ± 0.10	4.36	8.49	2.30	8.68 ± 0.16	27
**2t** ^e^	3-OH	2.12 ± 0.12	0.64	4.18 ± 0.36	0.32	0.43 ± 0.04	3.14	2.24	1.37	1.35 ± 0.08	61
**2u**	2-Cl	8.19 ± 0.17	0.08	0.89 ± 0.07	0.69	4.42 ± 0.35	0.14	4.50	0.30	0.61 ± 0.01	7

^a^ The CC_50_ values are the concentrations of compounds required to kill 50% of the cells. ^b^ The letters SI refer to the selectivity index. The SI figures are the ratios of the CC_50_ values of the compounds towards non-malignant HS-27 cells and the CC_50_ figures of a compound against a specific neoplastic cell line. ^c^ The letters PSE refer to the potency-selectivity expression. These figures are the product of the reciprocal of the average CC_50_ values against Colo205, HT-29 and CEM cells and the average SI value multiplied by 100. ^d^ Reference [16], ^e^ Reference [7], ^f^ Reference [30].

In regard to the sensitivity of the three neoplastic cell lines to the compounds in series **2**, the data in Table 4 revealed that 89% of the CC_50_ values are below 10 µM, 73% are below 5 µM and 21% have submicromolar CC_50_ values. The average CC_50_ values were also examined. The two most potent compounds are **2f** [2,5-(OCH_3_)_2_] and **2h** [3,4,5-(OCH_3_)_3_]. The dienone **2f** is 3 times more potent than the 3,4-dimethoxy analog **2e** while **2h** is 14 times more potent than **2g** [2,4,6-(OCH_3_)_3_].

The compounds in series **2** were also screened against Hs27 fibroblasts. The compounds with the highest CC_50_ values (>25 µM) are **2d**, **g**, **h**, **m**, **o**. The data in Table 4 reveal that the dimethoxy derivatives **2e** and **2f** are highly toxic to the fibroblasts (average CC_50_ value of 0.77 µM), while the trimethoxy analogs **2g**, **h** are 39 times less potent (the average CC_50_ value of 30.2 µM). Compounds with average SI values greater than 10 are **2d**, **h**, **m**, **o**. The large average SI value of 81.0 for **2h** is noteworthy. The PSE values of series **2** were computed and are listed in Table 4. Three compounds have PSE figures over 1000 namely **2h**, **m**, **o**. Thus, a number of lead molecules are described in this study and are listed in Table 5. Thus **2h** is a lead molecule in all four of the bioevaluations, while **2m** and **2o** are promising compounds in three of the bioassays.

**Table 5:** Identification of lead compounds based on the data in Table 4.

Bioevaluations	Promising Compounds
Average CC_50_ values	**2f**, **h**
Average SI values	**2d**, **h**, **m**, **o**
Low toxicity to normal cells	**2d**, **g**, **h**, **m**, **o**
PSE	**2h**, **m**, **o**

It is conceivable that the modes of action of **2a**–**u** towards the neoplasms are similar. In this case, the relative potencies of **2a**–**u** towards different neoplasms may be the same. In order to examine this possibility, use was made of Kendall’s coefficient of concordance [31], which is a statistical method for evaluating the similarity in rank between a collection of numbers. This methodology was applied to the CC_50_ values of **2a**–**u** against Ca9-22, HSC-2 and HSC-4 malignant cells. In this case, Kendall’s coefficient of concordance (W) is 0.7977, and a *p*-value of 0.0004 indicates the order of potency towards the neoplasms is the same. Since **2g**, **d**, **s** are outliers, plots were made omitting **2g** (W = 0.7668, *p* = 0.0010), **2g**, **d** (W = 0.7400, *p* = 0.0021) and **2g**, **d**, **s** (W = 0.7074, *p* = 0.0045). The analysis was repeated with the CC_50_ values of **2a**–**u** towards Colo205, HT-29 and CEM cells, which yielded a W value of 0.5834, *p* = 0.0200. The data generated when the outliers were removed are as follows: **2g** (W = 0.5324, *p* = 0.0475), **2g**, **d** (W = 0.4942, *p* = 0.0850) and **2g**, **d**, **s** (W = 0.4788, *p* = 0.1084). The effect of removing the outliers on the statistical results may be explained in the following ways. First, when one or more results are removed, statistical power is diminished. Second, Kendall’s coefficient of concordance deals with rank and is not concerned with potency differences. For example, **2g** is consistently weaker in potency compared to analogs.

In view of the relative potencies found using the Ca9-22, HSC-2 and HSC-4 assays and also using Colo205, HT29 and CEM screens, Kendall’s coefficient of concordance was applied using the CC_50_ values of the six neoplastic cell lines. The W value is 0.6086 and the p figure is 0.0000. The W and *p* values when outliers are systematically removed from consideration are as follows: 0.5533, 0.0000 (excluding **2g**), 0.5083, 0.0000 (excluding **2g**, **d**) and 0.4813, 0.0001(excluding **2g**, **d**, **s**). This observation has two utilities. First, it provides strong evidence that the modes of action of **2a**–**u** are similar. Second, one is justified in taking the average CC_50_ values of each compound in the pursuit of finding correlations between cytotoxic potencies and the physicochemical properties of the aryl substituents.

An attempt was made to find one or more physicochemical parameters that influence cytotoxic properties. Hence, linear and semilogarithmic plots were made between the average CC_50_ values of **2a**–**u** and the sigma/sigma star (σ/σ*), pi (π) and molecular refractivity (MR) values of the aryl substituents. These constants reflect the electronic, hydrophobic and steric properties, respectively, of the aryl groups. A summary of these results and the figures for the data generated are presented in the Supplemental Section. In a linear plot, a negative correlation was noted between the σ/σ* values and cytotoxic potencies (*p* = 0.02). No other correlations (*p* < 0.05) nor trends toward correlation (*p* < 0.10) were observed. Thus, potency increases as the magnitude of the electron-withdrawing properties of the aryl substituents rose. This observation may be due to the electron densities on the olefinic carbon atoms falling as the σ/σ* values rise, which increases the reactivity of the compounds towards cellular thiols, hence the extent of the cell death. Therefore, in the future, strongly electron-attracting groups should be placed in the aryl rings.

Attempts were made to find some of the ways that lead molecules exerted their cytotoxic properties. The average CC_50_ values of **2a**–**u** against Ca9-22, HSC-2 and HSC-4 cells are 0.70, 1.16 and 1.18 µM, respectively, indicating that Ca9-22 cells are more sensitive to the compounds in series **2** than are HSC-2 and HSC-4 cells. Hence the mode of action studies used Ca9-22 cells. Two lead compounds **2e** and **2r** were chosen for bio evaluation, as these molecules display excellent cytotoxic potencies and have the best average SI and PSE figures in series **2**.

Both **2e** and **2r** caused the mitotic arrest; i.e., the compounds inhibited various stages of mitosis and cell division. In contrast, a reference drug actinomycin D (AD) induced apoptotic bodies. These results are indicated in Figure 4.

A Western blot analysis was undertaken, and the results are presented in Figure 5. The enzyme poly(ADP-ribose)polymerase 1 (PARP 1) assists with the repair of damaged DNA. Thus, compounds which cleave PARP1 may be useful in cancer chemotherapy. As indicated in Figure 5, at a concentration of 3 µM, **2r** causes cleavage of PARP. Procaspase 3 is a precursor of caspase 3, and both of these enzymes are overexpressed in some tumours [32]. Hence reduction in the concentration of procaspase 3 and a rise in cleaved caspase 3 are useful properties for a candidate anticancer agent to demonstrate. At a concentration of 3 µM, **2r** causes a reduction in the concentration of procaspase 3 and, along with a concentration of 1 µM of **2e** and **2r,** shows procaspase 3 has been cleaved.

Another possible mode of action of the compounds in series **2** is their interfering with the cell cycle. The distribution of the Ca9-22 cells after treatment with **2e** and **2r** are given in Table 6. The data indicated **2e** and **2r** increase the percentage of cells in the subG1 phase, which contributes to the toxic effects of these compounds.

**Table 6:** Cell cycle distribution of Ca9-22 cells after treatment with **2e**, **r**.

Compound	Distribution (%) ^a^			
	SubG1	G1	S	G2/M
**2e** (0.3 µM)	2.2	33.1	20.5	43.7
**2e** (1 µM)	9.3 *	26.1	14.3	50.2
**2r** (1 µM)	11.5 *	18.5	12.0	57.9
**2r** (3 µM)	24.5 *	35.0	11.4	29.1
AD (1µM)	15.6 *	50.9	11.5	22.1
Control	1.7	33.3	17.0	47.7

Ca9-22 cells were treated for 24 h without (control) and with samples, and cell cycle distribution was measured. Representative cell cycle distributions in one of the triplicate samples are shown in Supplementary Figure S2. ^a^ Figures with an asterisk indicate a statistically significant difference from control (* *p* < 0.05).

**Scheme 1:**
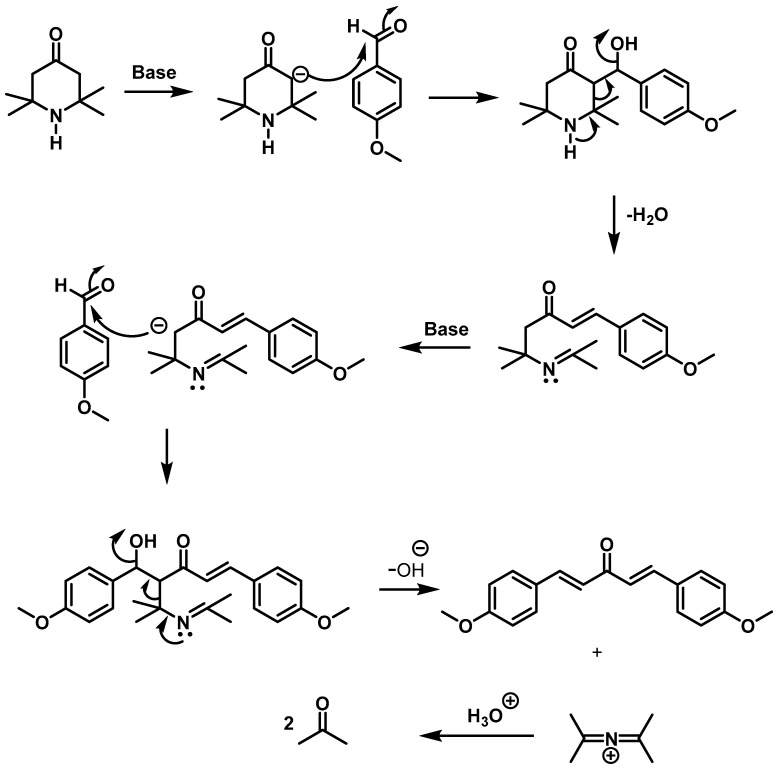
A possible reaction mechanism for the formation of series **3** from 2,2,6,6-tetramethyl-4-piperidone and aryl aldehydes.

There is a lack of coplanarity between the aryl rings and the adjacent olefinic groups. These interplanar angles θ are created between two carbon atoms of the aryl rings and the olefinic groups. These θ values are caused by non-bonded interactions between the ortho protons of the aryl ring and the equatorial hydrogen atom at locations 2 and 6 of the piperidine ring [33]. Hence, by replacing the protons at positions 2 and 6 of the piperidine ring with methyl groups, substantial increases in the sizes of the theta values may occur. The cytotoxic potencies of such compounds could be compared with the analogs in series **2**, which have the same aryl substituents. However, there was no reaction between 4-methoxybenzaldehyde and 2,2,6,6-tetramethyl-4-piperidone when the acid catalyzation method employed in preparing series **2** was used. Under basic conditions using sodium ethoxide or sodium methoxide, a product was isolated, which was identified as 1,5-bis(4-methoxyphenyl)-1,4-pentadien-3-one **3a**. A possible way in which this reaction takes place is presented in Scheme 1. In order to explore the generality of this reaction, the unsubstituted, 4-chloro and 4-fluoro analogs of **3a** (Figure 3)were prepared in yields of 22% to 41%. In the future, acylation of the piperidyl nitrogen atom of 2,2,6,6-tetramethyl-4-piperidone prior to attempted reactions with aryl aldehydes may lead to the formation of the desired products, since this molecular modification may prevent the opening of the piperidine ring.

## 5. Conclusions

**Figure 6:**
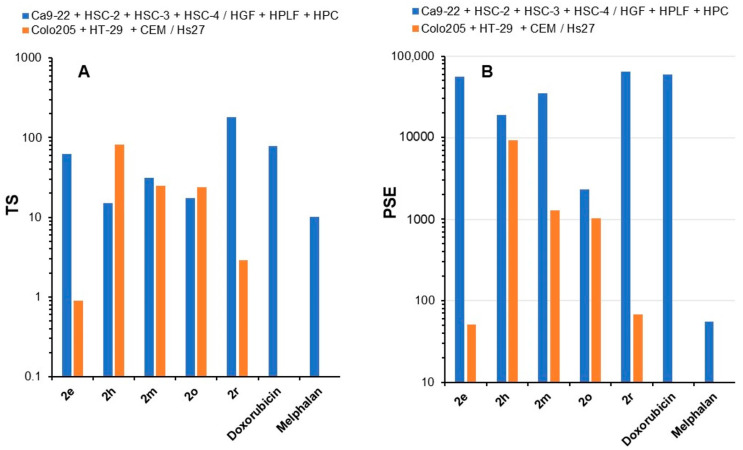
Comparison of SI and PSE values of 3,5-bis(benzylidene)-4-piperidones against OSCC (**A**) and colon and lymphoid leukemia cell lines (**B**). The SI and PSE values are derived from Table 1, Table 2 and Table 4. The cytotoxicity data of doxorubicin and melphalan against colon and lymphoid leukemia cell lines are not available.

This study revealed that, in general, the compounds in series **2** are highly toxic to a range of neoplastic cells. Most of these molecules have lower toxicity to various non-malignant cells. From these determinations, a number of lead compounds were identified. The SI and PSE values of possible lead compounds among 21 derivatives were plotted (Figure 6). This figure clearly shows that different sets of compounds showed higher tumor-specificity against different types of tumor cells: **2e** and **2r** for OSCC cell lines and **2h**, **2m** and **2o** for colon cancer and lymphoid leukemia cell lines. It should be noted that the tumor-specificity of **2e** and **2r** (SI = 62.1, 181) is comparable to that of doxorubicin (SI = 78.0) and slightly higher than that of melphalan (SI = 10.2) and four selected (3E,5E)-3,5-bis(arylidene)-4-piperidone (BAP) derivatives (SI = 12~18) [1].

Cytotoxic potencies increased as the electron-withdrawing properties of the aryl substituents were raised. However, the pi (π) and MR values of the aryl groups did not affect the magnitude of the CC_50_ values. In the future, analogs of series **2** will be prepared in which the substituents have increased electron-withdrawing properties, e.g., the 3-nitro-4-trifluoromethyl derivative (Σσ = 1.25 [17]). Further attempts will be made to introduce methyl groups at the 2 and 6 positions of the piperidine ring and to compare their cytotoxicity with the analogs in series **2**. 

## Data Availability

Not applicable.

## Supplementary Materials

The following is available online at https://www.mdpi.com/article/10.3390/medicines8120078/s1, 1. Percentage yields, melting points, ^1^H and ^13^C NMR spectra and mass spectra of **2a**–**u**. 2. Percentage yields, melting points, ^1^H NMR and mass spectra of **3a**–**d** and ^13^C NMR spectra of **3b**–**d**. Figure S1. Raw data of full Western blot of **2e**, **r**. Figure S2. Representative cell cycle distribution patterns in one of the triplicate samples in Table 5. 3. QSAR studies: Linear and Semilogarithmic determinations between the average CC_50_ values (in µM) and various physicochemical parameters in Tables S1 and S2. Linear and Semilogarithmic plots in Figures S3–S8.
