# Supplementary material for: Cytotoxic Tumour-Selective 1,5-Diaryl-3-Oxo-1,4-Pentadienes Mounted on a Piperidine Ring
Source: Medicines (Basel). 2021 Dec 16;8(12):78. doi: 10.3390/medicines8120078 (PMC8707244; doi:10.3390/medicines8120078)

SUPPLEMENTAL SECTION

Cytotoxic tumour-selective 1,5-diaryl-3-oxo-1,4-pentadienes mounted on a piperidine ring

Praveen K. Roayapalley[a,*], Hiroshi Sakagami[b], Keitaro Satoh[b], Shigeru Amano[b], Kenjiro Bandow[b], Renato J. Aguilera[c], Karla G. Cano Hernandez[c], Austre Y. Schiaffino Bustamante[c], Stephen G. Dimmock[d], Rajendra K. Sharma[e], Umashankar Das[a], Jonathan R. Dimmock[a]

1. Percentage yields, melting points, ^{1}H and ^{13}C NMR spectra, and mass spectra of **2a-u**…..S2.
2. Percentage yields, melting points, ^{1}H NMR and mass spectra of **3a-d** and ^{13}C NMR spectra of **3b-d.**………... S6.
3. Figure S1……………………………………………………………………………S7.
4. Figure S2……………………………………………………………………………S8.
5. QSAR…………………………………………………………………...………..S9.

1. Percentage yields, melting points, ^{1}H and ^{13}C NMR spectra and mass spectra of 2a-u.

*1.1 3,5-bis(benzylidene)-4-piperidone **(2a)***[1]

Yield: 89%; mp: 177-179 ˚C (lit 177-178 ˚C)[1]; ^{1}H NMR (500 MHz, DMSO-*d*6) δ ppm 4.25 (br s, 4 H) 7.44-7.54 (m, 10 H) 7.76 (s, 2 H). ^{13}C NMR (125 MHz, DMSO-*d*6) δ ppm 47.61, 128.67, 129.06, 130.47, 133.73, 134.92, 136.05, 187.70. MS (FD) *m/z* found: 275.1319, calculated *m/z*: 275.1310.

*1.2 3,5-bis(2-methoxybenzylidene)-4-piperidone **(2b)***

Yield: 68%; mp: 235-236 ˚C; ^{1}H NMR (500 MHz, DMSO-*d*6) δ ppm 3.87 (s, 6 H) 4.34 (s, 4 H) 7.08 (t, *J*=7.5 Hz, 2 H) 7.16 (d, *J*=8.2 Hz, 2 H) 7.26 - 7.34 (m, 2 H) 7.46 - 7.54 (m, 2 H) 8.03 (s, 2 H). ^{13}C NMR (125 MHz, DMSO-*d*6) δ ppm 44.06, 55.74, 111.61, 120.46, 122.24, 127.67, 130.22, 132.05, 135.11, 158.11, 182.44. MS (FD) *m/z* found: 335.1528, calculated *m/z*: 335.1521.

*1.3 3,5-bis(3-methoxybenzylidene)-4-piperidone **(2c)***[2]

Yield: 79%; mp: 195-197 ˚C (lit 194-196 ˚C)[2]; ^{1}H NMR (500 MHz, DMSO-*d*6) δ ppm 3.80 (s, 6 H) 3.99 (s, 4 H) 6.97 - 7.06 (m, 6 H) 7.38 (t, *J*=7.9 Hz, 2 H) 7.56 (s, 2 H). ^{13}C NMR (125 MHz, DMSO-*d*6) δ ppm 44.2, 55.1, 115.5, 115.7, 122.4, 128.8, 129.9, 134.8, 138.4, 159.2, 182.7. MS (FD) *m/z* found: 335.1519, calculated *m/z*: 335.1521.

*1.4 3,5-bis(4-methoxybenzylidene)-4-piperidone **(2d)***[3]

Yield: 84%; mp: 184-185 ˚C (lit182-184.6 ˚C)[3]; ^{1}H NMR (500 MHz, DMSO-*d*6) δ ppm 3.80 (s, 6 H) 3.96 (s, 4 H) 7.02 (d, *J*=8.8 Hz, 4 H) 7.44 (d, *J*=8.8 Hz, 4 H) 7.53 (s, 2 H). ^{13}C NMR (125 MHz, DMSO-*d*6) δ ppm 47.67, 55.29, 114.21, 127.55, 132.35, 133.38, 134.00, 159.87, 187.29. MS (FD) *m/z* found: 335.1512, calculated *m/z*: 335.1521.

*1.5 3,5-bis(3,4-dimethoxybenzylidene)-4-piperidone **(2e)***[3]

Yield: 73%; mp: 161-163˚C (lit: 162.2-165.4 ˚C)[3]; ^{1}H NMR (500 MHz, DMSO-*d*6) δ ppm 3.81 (d, *J*=1.3 Hz, 12 H) 4.01 (s, 4 H) 7.05 (s, 4 H) 7.08 (s, 2 H) 7.54 (s, 2 H). ^{13}C NMR (125 MHz, DMSO-*d*6) δ ppm 47.66, 55.52, 55.54, 111.59, 114.05, 123.90, 127.79, 133.88, 134.12, 148.50, 149.68, 187.24. MS (FD) *m/z* found: 395.1735, calculated *m/z*: 395.1733.

*1.6 3,5-bis(2,5-dimethoxybenzylidene)-4-piperidone **(2f)***[2]

Yield: 53%; mp: 136-137 ˚C (lit 133-137 ˚C)[2]; ^{1}H NMR (500 MHz, DMSO-*d*6) δ ppm 3.76 (s, 6 H) 3.81 (s, 6 H) 4.32 (br s, 4 H) 6.84 (br s, 2 H) 7.08 (br s, 4 H) 7.94 (br s, 2 H). ^{13}C NMR (125 MHz, DMSO-*d*6) δ ppm 44.04, 55.67, 56.08, 112.59, 115.74, 116.63, 122.84, 128.04, 135.06, 152.29, 152.73, 182.35. MS (FD) *m/z* found: 395.1735, calculated *m/z*: 395.1733.

1.7 3,5-bis(2,4,6-trimethoxybenzylidene)-4-piperidone ***(2g)***[3]

Yield: 43%; mp: 153-156 ˚C (lit: 152.3-156.1 ˚C)[3]; ^{1}H NMR (500 MHz, DMSO-d_6) δ ppm 3.38 (s, 4 H) 3.78 (s, 12 H) 3.81 (s, 6 H) 6.27 (s, 4 H) 7.36 (s, 2 H). ^{13}C NMR (125 MHz, DMSO-d_6) δ ppm 48.72, 55.42, 55.57, 90.64, 105.26, 126.47, 136.26, 158.87, 162.12, 187.51. MS (FD) *m/z* found: 455.1956, calculated *m/z*:455.1944.

1.8 3,5-bis(3,4,5-trimethoxybenzylidene)-4-piperidone ***(2h)***[3]

Yield: 76%; mp: 190-192 ˚C (lit: 193.7-194 ˚C)[3]; ^{1}H NMR (500 MHz, $CDCl_3$-*d*) δ ppm 3.82 (s, 12 H) 3.88 (s, 6 H) 4.52 (br s, 4 H) 6.52 (s, 4 H) 7.88 (s, 2 H) 10.31 (br s, 1 H). ^{13}C NMR (125 MHz, DMSO-d_6) δ ppm 43.99, 56.18, 60.23, 108.47, 127.09, 129.22, 139.51, 152.94, 182.20. MS (FD) *m/z* found: 455.1952, calculated *m/z*: 455.1944.

1.9 3,5-bis(benzo[d][1,3]dioxol-5-ylmethylene)-4-piperidone ***(2i)***[2]

Yield: 89%; mp: 220-222 ˚C (lit 218-220 ˚C)[2]; ^{1}H NMR (500 MHz, DMSO-d_6) δ ppm 3.95 (br s, 4 H) 6.09 (s, 4 H) 7.01 (d, *J*=0.9 Hz, 4 H) 7.05 (s, 2 H) 7.49 (s, 2 H). ^{13}C NMR (125 MHz, DMSO-d_6) δ ppm 48.13, 102.00, 109.06, 110.51, 126.28, 129.61, 134.08, 134.94, 148.08, 148.51, 186.54. MS (FD) *m/z* found: 363.1107, calculated *m/z*: 363.1107.

1.10 3,5-bis(2-fluorobenzylidene)-4-piperidone ***(2j)***[3]

Yield: 89%; mp: 140-142 ˚C (lit: 139.6-142.5 ˚C)[3]; ^{1}H NMR (500 MHz, DMSO-d_6) δ ppm 3.96 (s, 4 H) 6.79 - 6.92 (m, 6 H) 7.25 (t, *J*=7.9 Hz, 2 H) 7.47 (s, 2 H). ^{13}C NMR (125 MHz, DMSO-d_6) δ ppm 47.49, 115.65, 115.82, 122.48, 122.58, 124.56, 124.58, 125.82, 125.85, 131.09, 131.39, 131.46, 137.61, 159.44, 161.42, 187.17 MS (FD) *m/z* found: 311.1112, calculated *m/z*: 311.1122.

1.11 3,5-bis(3-fluorobenzylidene)-4-piperidone ***(2k)***

Yield: 84%; mp: 166-167 ˚C; ^{1}H NMR (500 MHz, DMSO-d_6) δ ppm 3.98 (s, 4 H) 7.22 - 7.28 (m, 2 H) 7.32 (d, *J*=7.6 Hz, 4 H) 7.48 - 7.53 (m, 2 H) 7.56 (s, 2 H). ^{13}C NMR (125 MHz, DMSO-d_6) δ ppm 44.15, 117.32, 117.62, 127.12, 129.46, 131.51, 136.41, 138.39, 161.00, 164.24, 182.79 MS (FD) *m/z* found: 311.1125, calculated *m/z*: 311.1122.

1.12 3,5-bis(4-fluorobenzylidene)-4-piperidone ***(2l)***[1]

Yield: 91%; mp: 209-211 ˚C (lit: 212-213 ˚C)[1]; ^{1}H NMR (500 MHz, DMSO-d_6) δ ppm 4.50 (s, 4 H) 7.31 - 7.45 (m, 4 H) 7.56 - 7.68 (m, 4 H) 7.87 (s, 2 H) 9.85 (br s, 2 H). ^{13}C NMR (125 MHz, DMSO-d_6) δ ppm 43.66, 115.98, 116.16, 127.70, 130.34, 133.13, 138.06, 161.83, 163.81, 182.32. MS (FD) *m/z* found: 311.1110, calculated *m/z*: 311.1122.

1.13 3,5-bis(3,4-difluorobenzylidene)-4-piperidone ***(2m)***

Yield: 77%; mp: 199-199 ˚C; ^{1}H NMR (500 MHz, DMSO-d_6) δ ppm 3.97 (s, 4 H) 7.33 - 7.38 (m, 2 H) 7.49 - 7.56 (m, 4 H) 7.60 (dd, *J*=12.0, 8.0 Hz, 1 H) 7.60 (dd, *J*=12.0, 8.0 Hz, 1 H). ^{13}C NMR (125 MHz, DMSO-d_6) δ ppm 47.29, 117.71, 119.36, 127.79, 131.71, 132.58, 136.77, 148.54, 150.62, 187.45 MS (FD) *m/z* found: 347.0932, calculated *m/z*: 347.0933.

1.14 3,5-bis(2,6-difluorobenzylidene)-4-piperidone ***(2n)***

Yield: 81%; mp: 196-198 ˚C; ^{1}H NMR (500 MHz, DMSO-d_6) δ ppm 3.60 (br. s., 4 H) 7.22 (t, *J*=8.2 Hz, 4 H) 7.37 (s, 2 H) 7.49 -7.60 (m, 2 H). ^{13}C NMR (125 MHz, DMSO-d_6) δ ppm 48.08, 112.22, 112.34, 112.53, 121.29, 152.37, 182.75 MS (FD) *m/z* found: 347.0947, calculated *m/z*: 347.0933.

1.15 3,5-bis(2-methylbenzylidene)-4-piperidone ***(2o)***

Yield: 91%; mp: 134-136 ˚C; ^{1}H NMR (500 MHz, DMSO-d_6) δ ppm 2.31 (s, 6 H) 3.81 (s, 4 H) 7.19 - 7.23 (m, 2 H) 7.24 - 7.32 (m, 6 H) 7.73 (s, 2 H). ^{13}C NMR (125 MHz, DMSO-d_6) δ ppm 19.63, 47.48, 125.72, 128.91, 129.24, 130.28, 132.50, 133.83, 136.07, 137.61, 187.68 MS (FD) *m/z* found: 303.1632, calculated *m/z*: 303.1623

1.16 3,5-bis(2-nitrobenzylidene)-4-piperidone hydrochloride ***(2p)***

Yield: 86%; mp: 220-221 ˚C; ^{1}H NMR (500 MHz, DMSO-d_6) δ ppm 4.23 (d, *J*=1.6 Hz, 4 H) 7.57 (d, *J*=7.6 Hz, 2 H) 7.74 - 7.79 (m, 2 H) 7.91 (td, *J*=7.6, 1.3 Hz, 2 H) 8.17 (s, 2 H) 8.29 (dd, *J*=8.4, 1.1 Hz, 2 H) 9.49 (br s, 1 H). ^{13}C NMR (125 MHz, DMSO-d_6) δ ppm 43.48, 125.29, 128.65, 129.62, 130.70, 130.80, 134.42, 137.51, 147.46, 182.18. MS (FD) *m/z* found: 366.1095, calculated *m/z*: 365.1012.

1.17 3,5-bis(4-hydroxy-3-methoxybenzylidene)-4-piperidone ***(2q)***

Yield: 74%; mp: 199-202 ˚C; ^{1}H NMR (500 MHz, DMSO-d_6) δ ppm 3.82 (s, 6 H) 3.99 (s, 4 H) 6.85 (br s, 2 H) 6.94 (dd, *J*=8.4, 1.7 Hz, 2 H) 7.04 (d, *J*=1.9 Hz, 2 H) 7.51 (s, 2 H). ^{13}C NMR (125 MHz, DMSO-d_6) δ ppm 44.03, 55.76, 115.57, 124.70, 124.76, 125.21,139.55, 147.74, 149.22, 181.89. MS (FD) *m/z* found: 367.1426, calculated *m/z*: 367.1420.

1.18 3,5-bis(3-hydroxy-4-methoxybenzylidene)-4-piperidone ***(2r)***

Yield: 79%; mp: 249 ˚C (decomposed); ^{1}H NMR (500 MHz, DMSO-d_6) δ ppm 3.85 (s, 6 H) 4.42 (s, 4 H) 6.96 (s, 2 H) 7.00 (dd, *J*=8.5, 1. 9 Hz, 2 H) 7.08 (d, *J*=8.5 Hz, 2 H) 7.71 (s, 2 H) 9.40 (br s, 2 H). ^{13}C NMR (125 MHz, DMSO-d_6) δ ppm 45.33, 55.69, 112.20, 117.15, 123.61, 125.94, 126.61, 138.85, 146.64, 149.67, 182.35. MS (FD) *m/z* found: 367.1433, calculated *m/z*: 367.1420.

1.19 3,5-bis(4-hydroxybenzylidene)-4-piperidone ***(2s)***[3]

Yield: 67%; mp >260 ˚C ((lit: 298.5 ˚C)[3]; ^{1}H NMR (500 MHz, DMSO-*d*$_6$) δ ppm 4.46 (s, 4 H) 6.89 - 6.93 (m, 4 H) 7.38 (d, *J*=8.5 Hz, 4 H) 7.78 (s, 2 H) 10.40 (br s, 1 H). ^{13}C NMR (125 MHz, DMSO-*d*$_6$) δ ppm 44.0, 116.0, 124.6, 124.7, 133.0, 139.1, 159.6, 182.0. MS (FD) *m/z* found: 307.1211, calculated *m/z*: 307.1208.

1.20 3,5-bis(3-hydroxybenzylidene)-4-piperidone ***(2t)***[2]

Yield: 39%; mp: 224-227 ˚C(lit: 226-230 ˚C)[2]; ^{1}H NMR (500 MHz, DMSO-*d*$_6$) δ ppm 3.94 (s, 4 H) 6.80 (dd, *J*=8.0, 1.7 Hz, 2 H) 6.83 (s, 2 H) 6.88 (d, *J*=7.9 Hz, 2 H) 7.23 - 7.27 (m, 2 H) 7.46 (s, 2 H). ^{13}C NMR (125 MHz, DMSO-*d*$_6$) δ ppm 48.14, 116.76, 117.30, 121.83, 130.10, 134.37, 135.86, 136.28, 158.03, 188.12. MS (FD) *m/z* found: 307.1213, calculated *m/z*: 307.1208.

1.21. 3,5-bis(2-chlorobenzylidene)-4-piperidone ***(2u)***

Yield: 83%; mp: 226-227 ˚C; ^{1}H NMR (500 MHz, DMSO-*d*$_6$) δ ppm 4.37 (d, *J*=1.9 Hz, 4 H) 7.46 - 7.55 (m, 6 H) 7.64 - 7.66 (m, 2 H) 8.00 (s, 2 H) 9.56 (br s, 1 H). ^{13}C NMR (125 MHz, DMSO-*d*$_6$) δ ppm 43.65, 127.57, 129.72, 130.00, 130.82, 131.63, 131.71, 134.11, 135.86, 182.24. MS (FD) *m/z* found: 344.0596, calculated *m/z*: 343.0531.

References:

1. Dimmock J. R, Padmanilayam M. P, Puthucode R. N, Nazarali A. J, Motaganahalli N. L, Zello G. A, Quail J. W, Oloo E. O, Kraatz H. B, Prisciak J. S. A conformational and structure-activity relationship study of cytotoxic 3,5-bis(arylidene)-4-piperidones and related N-acryloyl analogues. J. Med. Chem. 44 (2001) 586-593.
2. Gregory M, Dandavati A, Lee M, Tzou S, Savagian M, Brien K. A, Satam V, Patil P, Lee M. Synthesis, cytotoxicity, and structure–activity insight of NH- and N-methyl-3,5-bis-(arylidenyl)-4-piperidones. Med. Chem. Res. 22 (2013) 5588-5597.
3. Wu J, Zhang Y, Cai Y, Wang J, Weng B, Tang Q, Chen X, Pan Z, Liang G, Yang S. Discovery and evaluation of piperid-4-one-containing mono-carbonyl analogs of curcumin as anti-inflammatory agents. Bioorg. & Med. Chem. 21 (2013) 3058-3065.

2. Percentage yields, melting points, ^{1}H NMR and mass spectra of 3a-d and ^{13}C NMR spectra of 3b-d.

*2.1 (1E,4E)-1,5-bis(4-Methoxyphenyl)penta-1,4-dien-3-one **(3a)***[1]

Yield: 37%; mp: 120-122 °C (lit 121-122°C)[1]; ^{1}H NMR (500 MHz, DMSO-d_6) δ ppm 3.80 (s, 6 H) 7.01 (d, *J*=8.8 Hz, 4 H) 7.20 (s, 1 H) 7.17 (s, 1 H) 7.71 (s, 1 H) 7.72 - 7.75 (m, 5 H). MS (FD) *m/z* found: 294.1271, calculated *m/z*: 294.1256.

*2.2 (1E,4E)-1,5-Diphenylpenta-1,4-dien-3-one **(3b)***[2]

Yield: 22 %; mp: 110-112 °C (lit 110-112 °C)[2]; ^{1}H NMR (500 MHz, DMSO-d_6) δ ppm 7.09 (d, *J*=15.9 Hz, 2H), 7.37 – 7.46 (m, 6H), 7.58 – 7.65 (m, 4H), 7.74 (d, *J* =15.9 Hz, 2H). ^{13}C NMR (125 MHz, $CDCl_3$) 125.69, 128.62, 129.21, 130.74. 135.81, 143.56, 189.11. MS (FD) *m/z* found: 234.1057, calculated *m/z*: 234.1045.

*2.3 (1E,4E)-1,5-bis(4-Chlorophenyl)penta-1,4-dien-3-one **(3c)***[1]

Yield: 41%; mp: 192-193 °C (lit 192-193 °C)[1]; ^{1}H NMR (500 MHz, $CDCl_3$) δ ppm 7.04 (d, *J*=15.9 Hz, 2 H), 7.40 (dd, *J*=8.6 Hz, 4 H), 7.56 (d, *J*=8.6 Hz, 4 H), 7.70 (d, *J*=15.9 Hz, 2 H). ^{13}C NMR (125 MHz, $CDCl_3$) δ 126.0, 128.7, 128.7, 129.3, 129.3, 133.3, 136.5, 142.1, 188.3. MS (FD) *m/z* found: 302.0279, calculated *m/z*: 302.0265.

*2.4 (1E,4E)-1,5-bis(4-Fluorophenyl)penta-1,4-dien-3-one **(3d)***[2]

Yield: 29%; mp: 146-148 °C (lit 150-152° C)[2]; ^{1}H NMR (500 MHz, $CDCl_3$) δ ppm 7.00 (d, *J*=15.9 Hz, 2 H), 7.11 (dd, *J*=8.7 Hz, 4 H), 7.60 (d, *J*=8.7 Hz, 4 H), 7.71 (d, *J*=15.9 Hz, 2 H). ^{13}C NMR (125 MHz, $CDCl_3$) δ 115.8, 125.4, 130.8, 141.5, 161.6, 164.9, 188.3 MS (FD) *m/z* found: 270.094, calculated *m/z*: 270.085.

References:

1. Wei X, Du Z.-Y, Zheng X, Cui X.-X, Conney A. H, Zhang K. Synthesis and evaluation of curcumin-related compounds for anticancer activity. Eur. J. Med. Chem. 53 (2012) 235-245.

2. Weber W. M, Hunsaker L. A, Roybal C. N, Bobrovnikova-Marjon E. V, Abcouwer S. F, Royer R. E, Deck L. M, Vander Jagt D. L. Activation of NFκB is inhibited by curcumin and related enones. Bioorg. & Med. Chem. 14 (2006) 2450-2461.

Figure S1. Raw data of full Western blot of **2e**,**r**. Protein(9 µg) was loaded to each lane.
lane 1: control
lane 2: actinomycin D (1 µM)
lane 3: **2e** (0.3 µM)
lane 4: **2e** (1 µM)
lane 5: **2r** (1 µM)
lane 6: **2r** (3 µM)

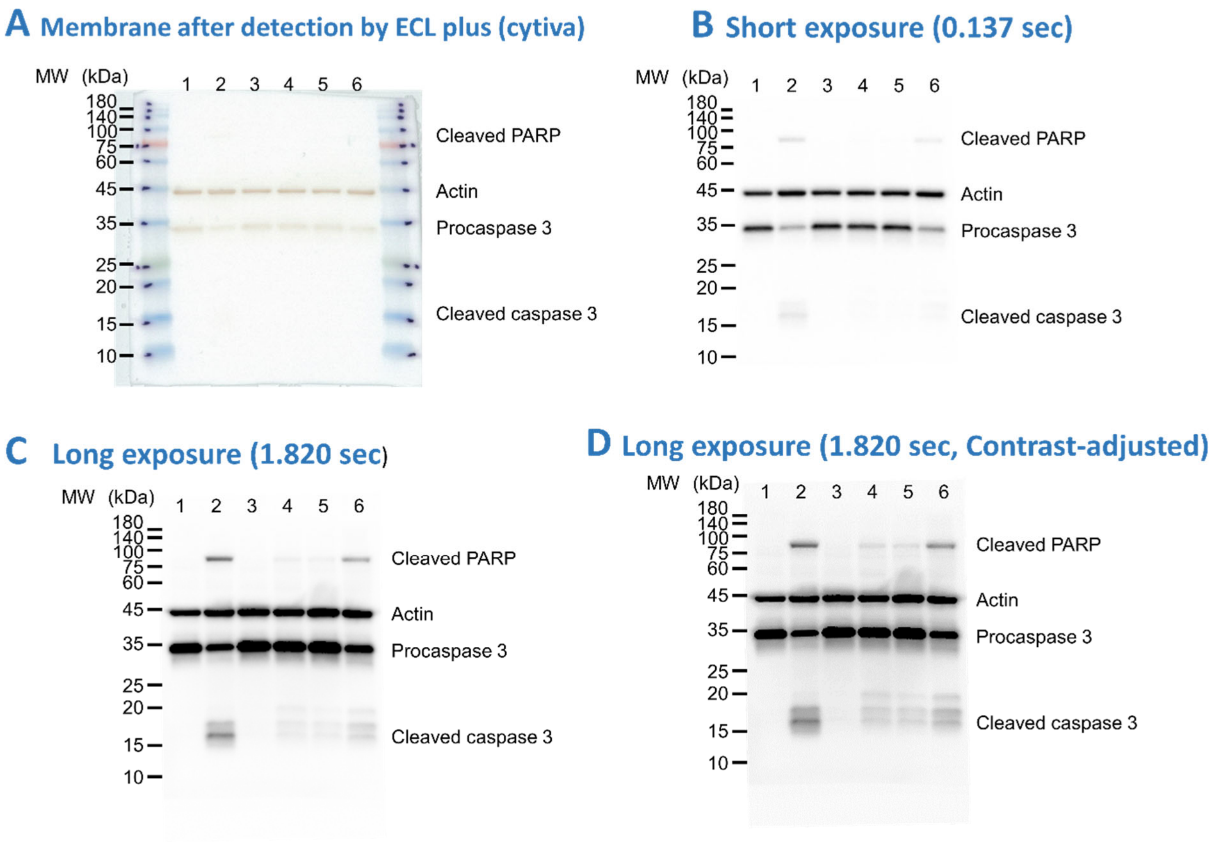

Figure S2. Representative cell cycle distribution patterns in one of triplicate samples in Table 5. Ca9-22 cells were incubated for 24 h without (control) or with the indicated concentrations of test compounds (upper panel). Pictures of morphologies of the cells before cell harvest are shown in lower panel. Reproducible morphological changes were confirmed.

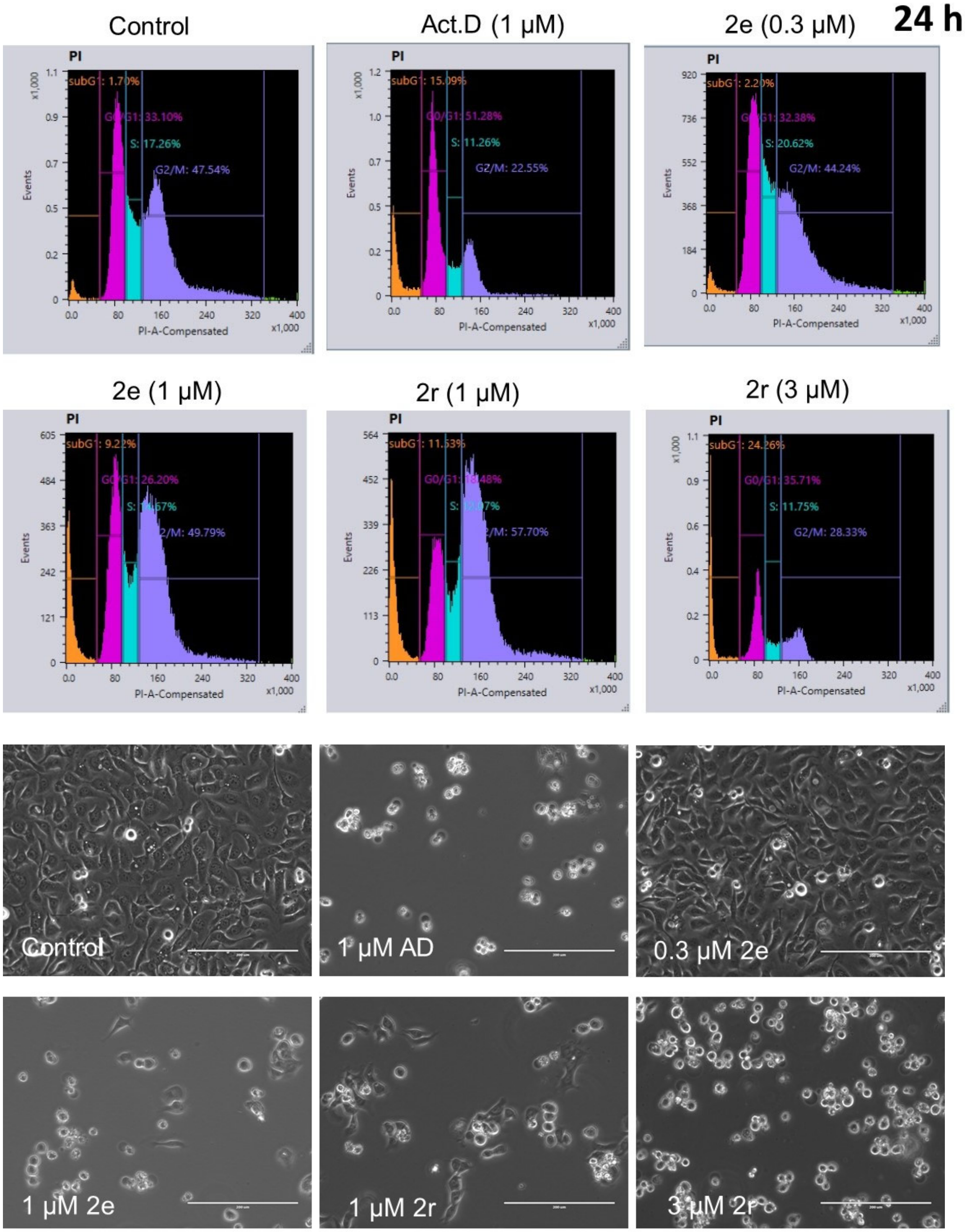

QSAR.

Linear and semilogarithmic plots were made between the σ/σ*, π and MR values of the aryl substituents in **2a-u** and the average CC_{50} values. A summary of the results are presented in Tables S1 and S2. In addition, the six plots which produced these data are portrayed in Figures S3-S8 of this supplementary section.

Table S1. Linear determinations between the average CC_{50} values (in μM) and various physicochemical parameters.

Physicochemical parameter	p value	Correlation (+ or -)
σ/σ*	0.021	Negative correlation
π	0.464	Negative correlation
MR	0.326	Positive correlation

Table S2. Semilogarithmic determinations between the average CC_{50} values (in μM) and various physicochemical parameters.

Physicochemical parameter	p value	Correlation (+ or -)
σ/σ*	0.170	Negative correlation
π	0.441	Negative correlation
MR	0.689	Negative correlation

Figure S3. Linear determinations between the average CC_{50} values (in µM) and σ/σ*

Correlations			
		Average CC_{50} (µM)	σ/σ*
Average CC_{50} (µM)	Pearson Correlation	1.000	-0.501
	Sig. (2-tailed)		0.021
	N*	21	21
σ/σ*	Pearson Correlation	-0.501	1.000
	Sig. (2-tailed)	0.021	
	N*	21	21

*N= Number of samples

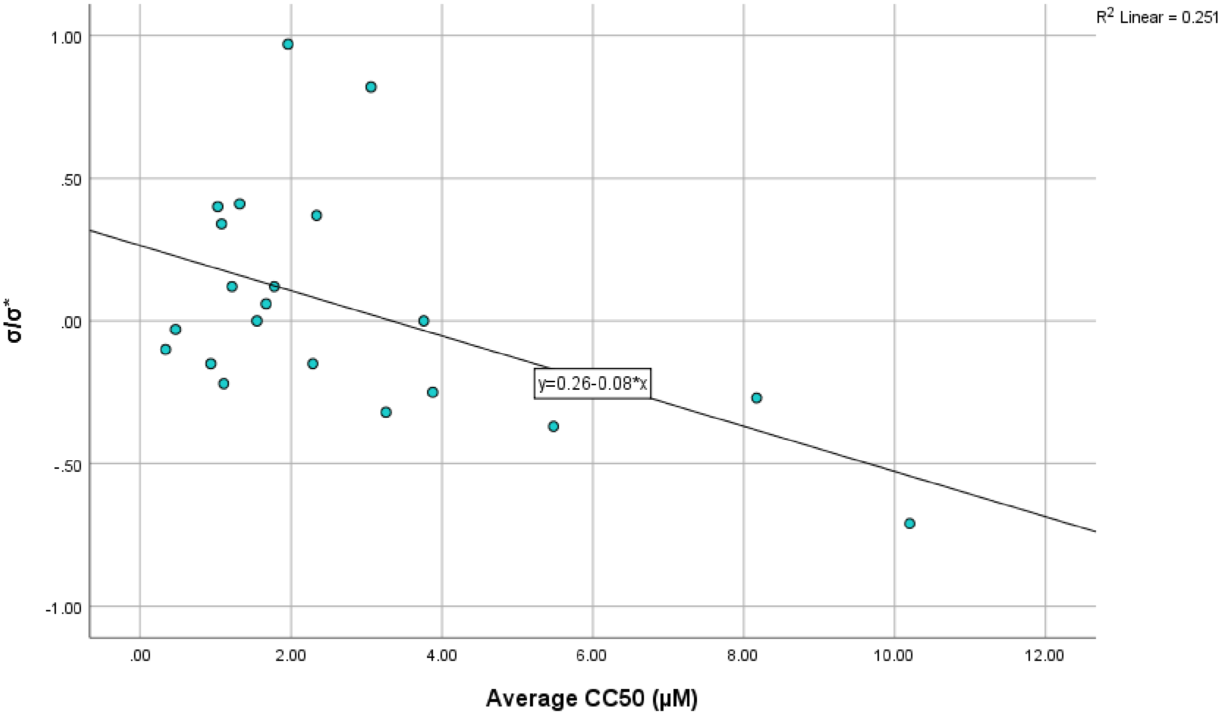

Figure S4. Linear determinations between the average CC_{50} values (in µM) and π.

Correlations			
		π	Average CC_{50} (µM)
π	Pearson Correlation	1.000	-0.169
	Sig. (2-tailed)		0.464
	N*	21	21
Average CC_{50} (µM)	Pearson Correlation	-0.169	1.000
	Sig. (2-tailed)	0.464	
	N*	21	21

*N= Number of samples

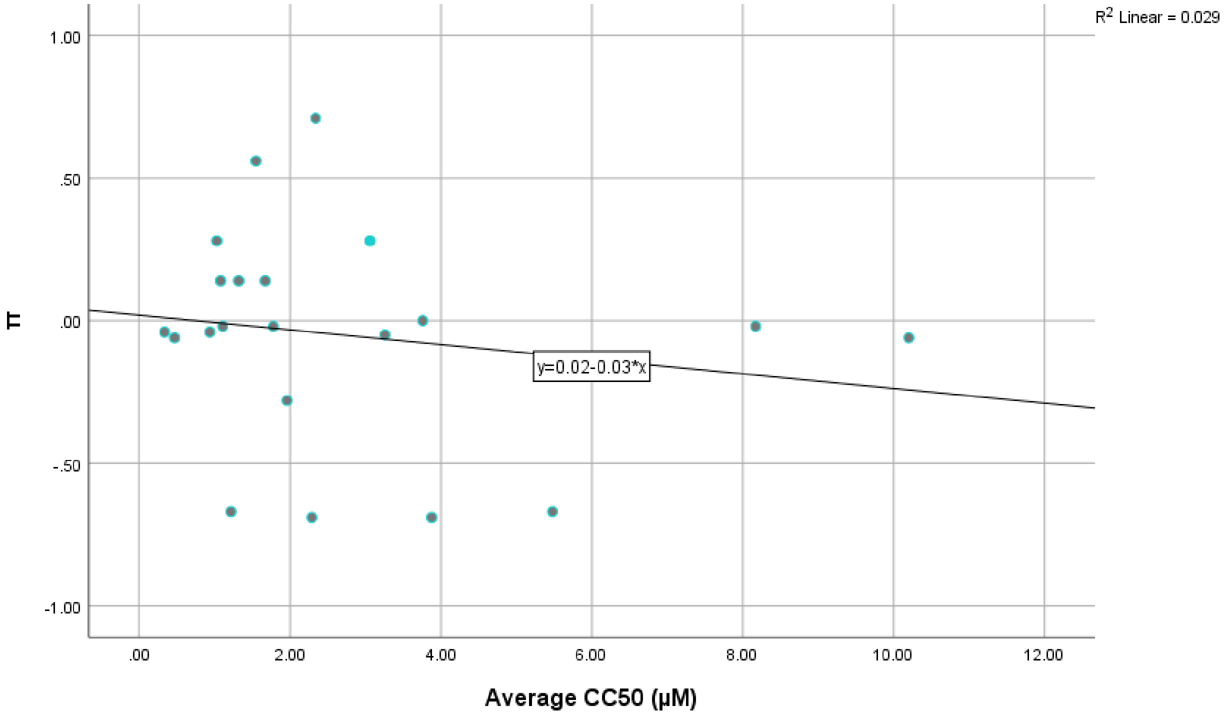

Figure S5. Linear determinations between the average CC_{50} values (in µM) and MR.

Correlations			
		Average CC_{50} (µM)	MR
Average CC_{50} (µM)	Pearson Correlation	1.000	0.225
	Sig. (2-tailed)		0.326
	N*	21	21
MR	Pearson Correlation	0.225	1.000
	Sig. (2-tailed)	0.326	
	N*	21	21

*N= Number of samples

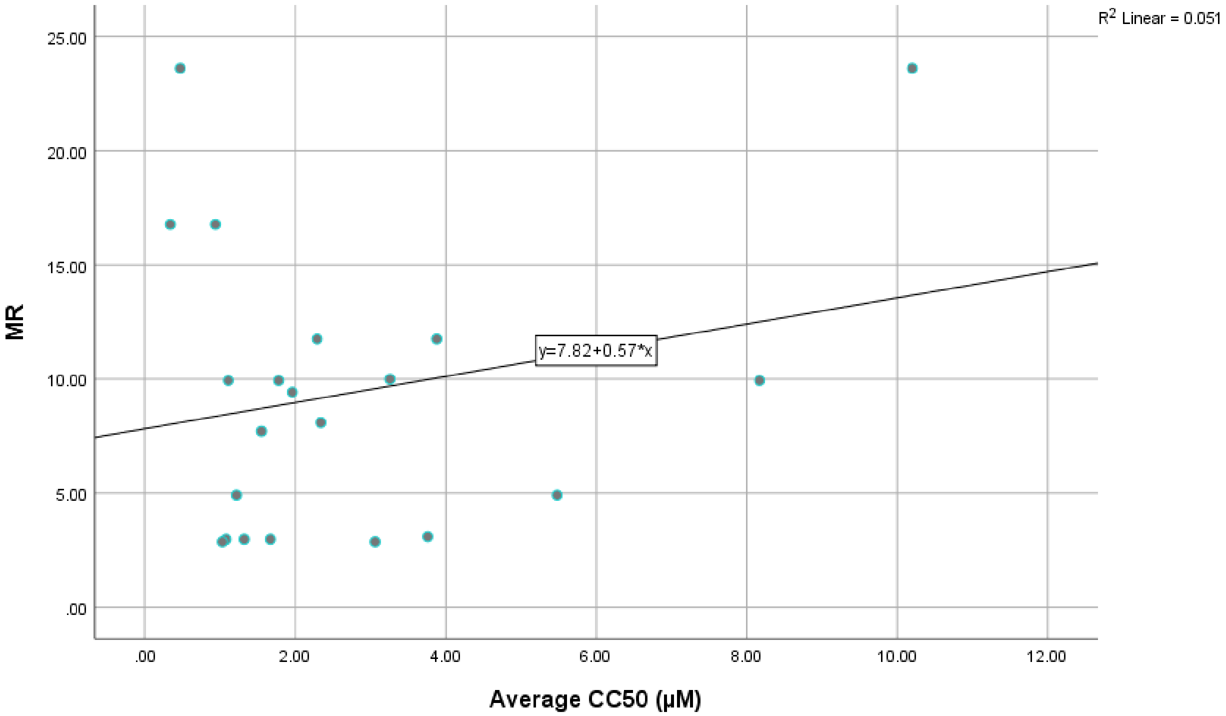

Figure S6. Semilogarithmic determinations between the average CC_{50} values (in µM) and σ/σ*

Correlations			
		σ/σ*	Log[Avg CC_{50} (µM)]
σ/σ*	Pearson Correlation	1.000	-0.311
	Sig. (2-tailed)		0.170
	N*	21	21
Log[Avg CC_{50} (µM)]	Pearson Correlation	-0.311	1.000
	Sig. (2-tailed)	0.170	
	N*	21	21

*N= Number of samples

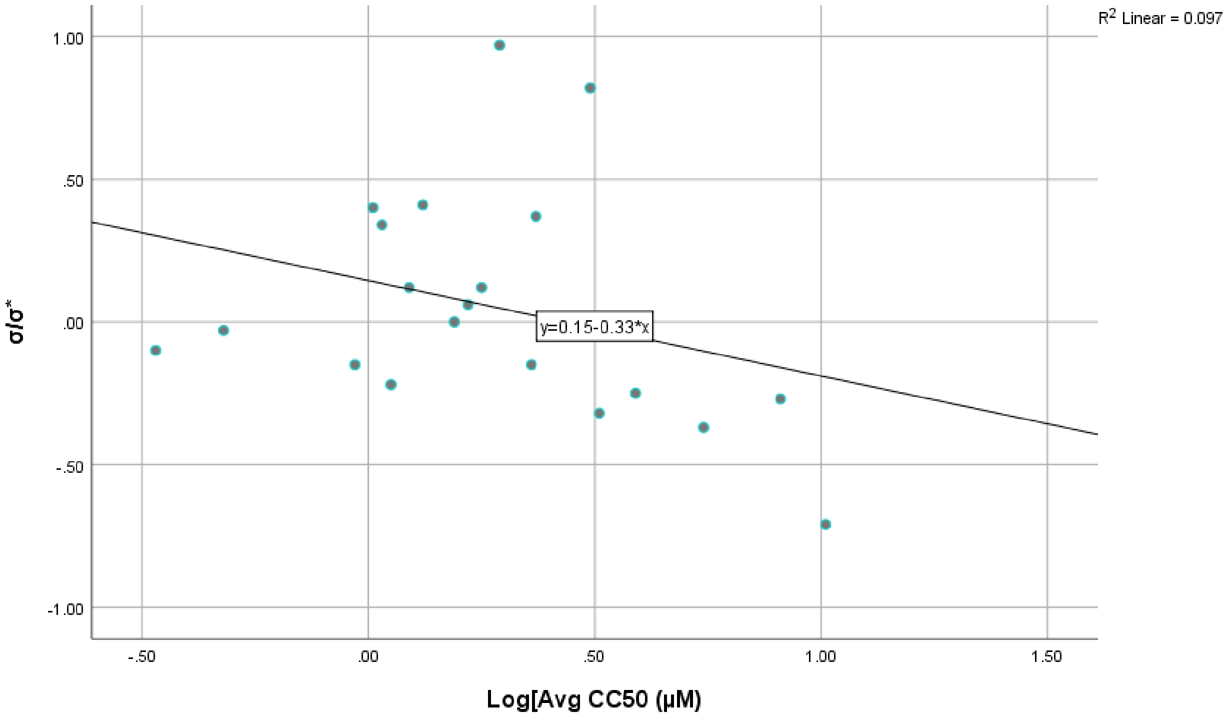

Figure S7. Semilogarithmic determinations between the average CC_{50} values (in μM) and π.

Correlations

		Log[Avg CC_{50} (μM)]	π
Log[Avg CC_{50} (μM)]	Pearson Correlation	1.000	-0.178
	Sig. (2-tailed)		0.441
	N*	21	21
π	Pearson Correlation	-0.178	1.000
	Sig. (2-tailed)	0.441	
	N*	21	21

*N= Number of samples

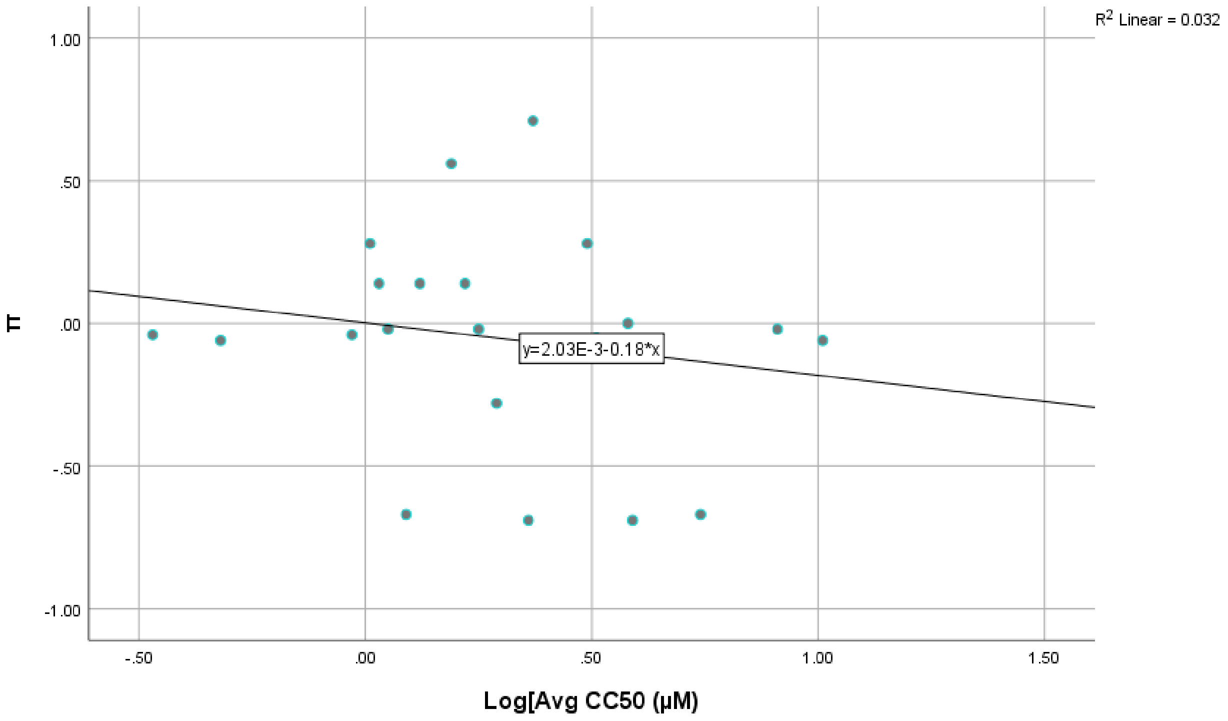

Figure S8. Semilogarithmic determinations between the average CC_{50} values (in µM) and MR.

Correlations

		Log[Avg CC_{50} (µM)]	MR
Log[Avg CC_{50} (µM)]	Pearson Correlation	1.000	-0.093
	Sig. (2-tailed)		0.689
	N*	21	21
MR	Pearson Correlation	-0.093	1.000
	Sig. (2-tailed)	0.689	
	N*	21	21

*N= Number of samples

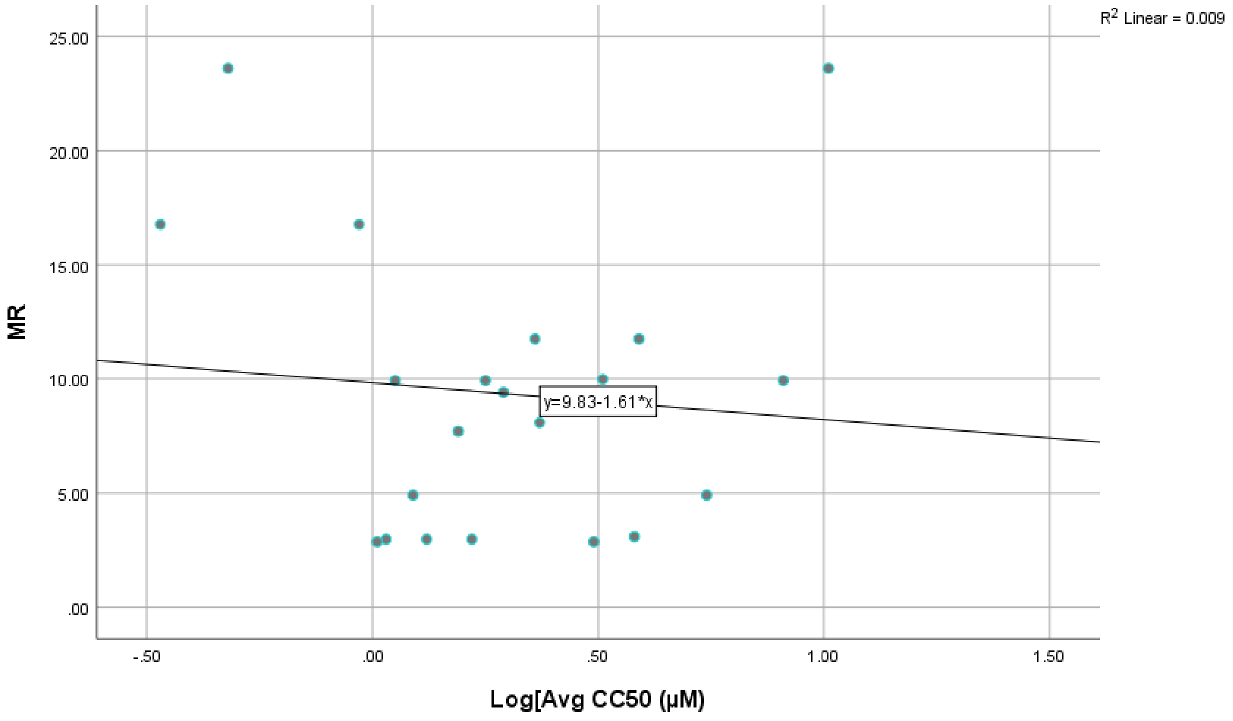

Supplement: Supplementary file 1 [file medicines-08-00078-s001.zip › medicines-1425704-supplementary.pdf]
